# Supplementation of microencapsulated probiotics modulates gut health and intestinal microbiota

**DOI:** 10.1002/fsn3.3414

**Published:** 2023-05-10

**Authors:** Ishwari Gyawali, Guilian Zhou, Guli Xu, Genghui Li, Yujun Wang, Yuxian Zeng, Jincheng Li, Jingjing Zhou, Canjun Zhu, Gang Shu, Qingyan Jiang

**Affiliations:** ^1^ Guangdong Laboratory for Lingnan Modern Agriculture, College of Animal Science South China Agricultural University Guangzhou China; ^2^ Quality Control for Feed and Products of Livestock and Poultry Key Laboratory of Sichuan Province Chengdu China

**Keywords:** gut health, *Lactobacillus paracasei*, mice, microbiota, microencapsulation, polyacrylate resin

## Abstract

The beneficial effect of probiotics on host health is impaired due to the substantial loss of survivability during gastric transit caused by small intestinal enzymes and bile acids. Encapsulation helps to preserve the probiotics species from severe environmental factors. *Lactobacillus paracasei*, highly sensitive probiotic species to gastric acid, was encapsulated with polyacrylate resin. C57BL/6 male mice were equally divided into three groups; control group was fed with basal diet without any additives, the un‐encapsulated group was fed with 0.1% of a mixture of encapsulating material and *L. paracasei*, and encapsulated group was fed with 0.1% encapsulated *L. paracasei* (microcapsule) for 4 weeks. The result showed elevated fecal moisture percentage in the encapsulated group, but not in the un‐encapsulated group. Further study showed that the ratio of villus height to crypt depth in the small intestine was significantly higher compared to un‐encapsulated and the control group. Microencapsulated probiotics also remarkably increased intestinal mucin and secretory immunoglobulin A (sIgA) concentration, intestinal MUC‐2, and tight junction protein mRNA expression levels improving the intestinal barrier function of mice. In addition, microcapsules also reduced proinflammatory factor mRNA expression, while considerably increasing anti‐inflammatory factor mRNA expression. Microbiota metabolites, fecal LPS (Lipopolysaccharide) were downregulated, and acetate and lactate were upraised compared to control. Furthermore, glutathione peroxidase (GSH‐Px) and TAOC levels were increased and Malondialdehyde (MDA) was decreased improving antioxidant capacity. Microflora and bioinformatic predictive analysis of feces showed that encapsulated probiotics remarkably increased *Lactobacillus* proportions. Mice's intestinal health can thus be improved by using microencapsulated probiotics.

## Graphical abstract

We employed a new protective material to create microcapsules that safeguard probiotic strains against harsh environmental conditions. Our findings demonstrate that the microcapsules we developed have a positive effect on the gut health of mice.

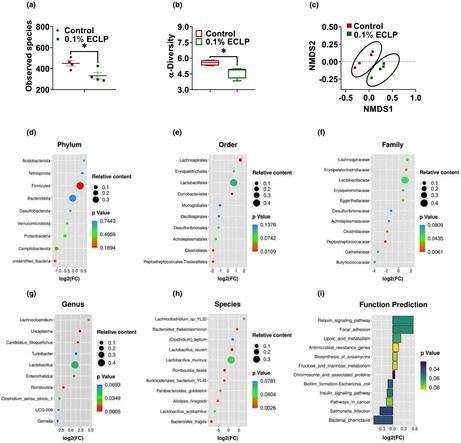

## 1 INTRODUCTION

Intestine helps in food digestion and absorption of food‐derived nutrients in the host (Okumura & Takeda, 2017). The three components of the intestine, named single‐cell layer epithelium, microbiome, and immune system, together have a vital role in nourishing homeostasis in host health (Fay et al., 2017). Intestinal epithelial cells contribute to the maintenance of host‐microbe symbiosis by controlling the nutrient uptake and protecting against stress (Bonis et al., 2021; Fay et al., 2017). Gut microbiota refers to the community of microorganisms that inhabit the digestive tract (Turner, 2018) and the microbial environment is dominated by bacteria, gram‐positive *Firmicutes*, and gram‐negative *Bacteroidetes*. The diversity of the microbiome is closely associated with intestinal health (Rinninella et al., 2019). The enteric microbiota, inhabiting the gastrointestinal tract, has a significant contribution to nutrient and drug metabolism, detoxification, and prevention of the pathogens' colonization along with the induction and regulation of essential components of the host innate and adaptive immune system (Jandhyala et al., 2015; Magne et al., 2020). Meanwhile, the immune system organizes the principal aspect of the symbiotic relationship between the host and highly diversified microorganisms. But, the changes in the composition of the gut microbiota or disruption of the interaction between host microbes and the immune system affect intestinal health and can lead to the development of autoimmune diseases or disorders (Zheng et al., 2020). Thus, the maintenance of the gut microbiota is crucial to regulate immune homeostasis and impart health benefits to the host.

The use of antibiotics, shifts in diet, age, or infection can disturb the gut microbiota leading to pathogenic, inflammatory, and metabolic conditions (Hasan & Yang, 2019; Walker & Lawley, 2013) Various approaches have been suggested to regulate the composition of the microbial community, which includes inoculation of probiotics (Hasan & Yang, 2019), prebiotics (Yue et al., 2020), oligosaccharides, dietary fiber (Cheng et al., 2017), traditional Chinese medicines (R. Zhang et al., 2020), fecal microbiota transplantation (FMT) (Gupta et al., 2020), etc.

Probiotics are live microorganisms, when administered in sufficient numbers enhance the host's health. They play a substantial role in intestinal health by restoring gut microbiome composition and providing a favorable environment for the commensal bacteria that results in the treatment of many infections (Anselmo et al., 2016; Wang et al., 2021). But, the probiotics strain selection is limited as they lack stability during storage, transportation, and gastric transit. Higher temperature, oxygen level, and relative humidity (RH) are harmful to many probiotics (Yao et al., 2020). Similarly, when probiotics are ingested, it needs to face harsh environmental complexity in the gastrointestinal (GI) tract. Generally, they can survive at the pH range of about 6–7 (Yeung et al., 2016) but, gastric fluids are highly acidic (pH around 1–3) that can be deleterious to probiotic species (Sarao & Arora, 2017). In addition, high ionic strength and enzyme (pepsin) activity in the stomach (Yao et al., 2020; Yeung et al., 2016), bile acid, and digestive enzymes (lipases, proteases, and amylases) in the small intestine also affect the viability of probiotics (Han et al., 2021; Yao et al., 2020). As a result, there is a reduction in the number of bacteria reaching the hindgut. Thus, several methods have been documented to surmount these obstacles and enhance the ability to survive in the gastrointestinal tract, strengthen mucoadhesion characteristics, and elevate colonization (Terpou et al., 2019).

Microencapsulation has been recommended as a propitious solution to solve these issues regarding its viability and potentiality (Pupa et al., 2021; Šipailienė & Petraitytė, 2018; Vivek et al., 2023). Microencapsulation protects the probiotics from environmental stress, GI tract insult and holds its structure in the upper GI tract before releasing it in the intestinal area, enhancing its efficacy (Wang et al., 2020; Yeung et al., 2016). This technology has been widely used in clinical medicine for the controlled release of encapsulated drugs (Lopez‐Mendez et al., 2021). However, the material used for microencapsulation should be selected wisely. Some encapsulating material may not break down on the targeted site and others can dissolve partially, preventing the complete release of probiotic species into the hindgut, which could excrete out without utilization (Lee et al., 2019). Considering these facts, we developed a microcapsule to deliver the probiotics in the intestine for its efficient utilization. *Lactobacillus paracasei* was taken as a probiotic species encapsulated with Polyacrylate resin to prepare a microcapsule, used as a feed additive. *L. paracasei* is an extensively used probiotic strain, but it is highly sensitive to low pH (Shori, 2017). Polyacrylate resin is pH‐sensitive material that can dissolve in the intestinal juice of pH ≥7; thus, it can protect *L. paracasei* from gastric acid, release it on the lower GI tract, and enhance its potential as probiotics. Nevertheless, there is no research using this matter as encapsulating material for probiotics, although it is used as adhesive material. Thus, our objective was to enhance the survivability of *L. paracasei* probiotics by utilizing microencapsulation, which shields them from harmful effects of gastric acid and other gastrointestinal insults, while also studying the effect of the prepared microcapsules on the morphology, immune response, antioxidant capacity, and gut microbiota of mice.

## 2 MATERIALS AND METHODS

### 2.1 Microencapsulation of probiotics

First, starch pellets are used as a carrier and added to the coating pot which was preheated to 37°C. A probiotic bacterium known as *L. paracasei* GDMCC 1.649, which was isolated from the human gut, was used for microencapsulation. After a 10‐min preheating period, the bacterial liquid (*L. paracasei*) was sprayed onto the pellets using a spray bottle. The purpose of this step was to ensure that the bacteria are evenly distributed on the surface of the starch pellets.

Once the bacterial liquid was applied, the enteric coating material was then sprayed onto the pellets. This coating material is pH‐dependent, it will remain intact as it passes through the stomach and will only dissolve in the intestines where the pH is higher. Polyacrylic resin is commonly used as an enteric coating material due to its pH sensitivity and ability to protect the bacteria until it reaches the intestines.

By encapsulating the bacteria in starch pellets and then applying an enteric coating, the survivability of the probiotics is increased. This process protects the bacteria from the harsh acidic environment of the stomach and ensures that they can reach the intestines intact, where they can provide their beneficial effects.

### 2.2 In vitro test of coated probiotics

The artificial gastric juice (Yuanye Bio‐Technology Co Ltd, R30386) and small intestine fluid (Yuanye Bio‐Technology Co Ltd, R30384) were prepared according to the manufacturer's instructions. The coated and uncoated bacteria were inoculated into separate Petri dishes of the artificial gastric juice and then incubated at a temperature of 37°C for 1.5 h. Following this, the bacteria were moved from the artificial gastric juice to separate Petri dishes of the artificial small intestine fluid and incubated again at 37°C for another 1.5 h. After spending 1.5 h in the artificial small intestine fluid, the bacterial solution was diluted by a factor of 10,000. This diluted bacterial solution was then plated on a suitable agar medium and incubated under appropriate conditions to promote the formation of colonies. Finally, the number of monoclonal colonies that formed was counted, and a comparison between the number of colonies that formed from the coated and uncoated bacteria was performed to determine whether the coating provided any protection against the simulated digestive fluids.

### 2.3 Animals, experimental design, and dietary treatments

A total of 30 C57BL/6 male mice (5 weeks) were obtained from the Animal Experiment Center of Guangdong Province (Guangzhou, Guangdong, China) and were caged individually, in a sterile and controlled environment with a temperature of a 24°C ± 2°C, relative humidity of 65% ± 10% and 12 h light 12 h/dark cycle. For 2 weeks, mice were provided with mouse feed and water ad libitum. After 2 weeks of adaptation, mice were assigned into three groups (*n* = 10) according to their weight: one group was blank control (Control) which received a basal diet without any additives, the second group was un‐encapsulated *L. paracasei* (0.1% MELP), which received 1 g/Kg of a mixture of *L. paracasei* GDMCC 1.649 (0.25 g/Kg) and coating material, polyacrylate resin (0.75 g/Kg), and the last group was 0.1% encapsulated *L. paracasei* (0.1% ECLP) which received 1 g/Kg of encapsulated *L. paracasei*. Here, encapsulated *Lactobacillus* was prepared by encapsulating *L. paracasei* GDMCC 1.649 with polyacrylate resin by Hefei Ansheng Pharmaceutical Technology Co., Ltd, Hefei, China. Feed was prepared with water by maintaining a pH of 3.5 to protect encapsulating material from disintegration in the presence of water. The animals were individually housed in cages. Each animal's body weight and feed intake were recorded weekly. The Nuclear magnetic resonance system (Body Composition Analyzer MiniQMR23‐060H‐I, Niumag, China) was used to measure the body composition.

### 2.4 Fecal sample collection

For moisture content, fresh feces were collected every day for a week and then once a week for 3 weeks. Feces were collected instantly after ejection and placed on airtight tubes to prevent evaporation. The collected fresh feces were weighed in a tube, dried for 24 h at a 60°C dry oven, and reweighted to get dry weight. At the end, the moisture content was determined as (weight of feces before drying − weight of feces after drying)/weight of feces before drying × 100%. Similarly, all the feces were also collected regularly from the litter on 10 mL tubes to measure total dry fecal weight and use the feces for further studies. Feces collected in sealed tubes (10 mL) were dried for 48 h at 37°C to protect them from the loss of volatile nutrients that can be evaporated and lost at a higher temperature. After drying, feces were weighed to obtain dry fecal weight and stored at −80°C for later use.

### 2.5 Sample collection and processing

After feeding for 28 days, all mice were sacrificed. Blood was harvested from an eyeball. Blood samples were collected in a 1.5 mL centrifuge tube, placed for 1 h at room temperature for clotting, and centrifuged (3500 rpm, 15 min, and 4°C). The serum of each sample was separated and stored at −20°C for subsequent detection and analysis. From the dissected mice, tissues and internal organs were observed and weighed. Intestinal tissues and their contents were stored at −80°C for future use. The parts of separated specimens of the intestinal section (duodenum, jejunum, and ileum) were fixed in a 4% paraformaldehyde solution for morphological study.

### 2.6 Morphological study

The specimens placed in paraformaldehyde were embedded in paraffin wax, and slices were sectioned at 5 μm. The obtained sections were stained with hematoxylin and eosin (HE) by mounting in a glass slide. Afterward, the slides were observed under the microscope (Olympus, Tokyo, Japan) for villi length (V) and crypt depth(C). The measurement of V and C was done using Image (Image‐Pro Plus 6.1 Media Cybernetics, Rockville, MD, USA). Finally, the ratio of V and C (V/C) was determined.

### 2.7 Western blotting

The protein was extracted from intestinal tissues using RIPA lysis buffer (P0013B, Beyotime), and its concentration was determined using a BCA protein assay kit (23227, Thermo Scientific). The protein concentration was then adjusted to 20 μg/20 μL and denatured with protein loading buffer (LT 101, EpiZyme) by boiling it in water for 10 min. The western blot (WB) procedures followed a previous study (Yuan et al., 2020). The primary antibody used was anti‐Claudin (sc‐166338, 1:1000, Santa Cruz) and β‐Tubulin was used as the loading control. The proteins were visualized on a PVDF membrane using Protein Simple (Santa Clara, CA USA) and super ECL Enhanced Pico Light Chemiluminescence Kit (SQ 101, EpiZyme). The protein expression level was analyzed using ImageJ (National Institutes of Health, USA).

### 2.8 Biochemical indices

The level of inflammatory factor (IL‐1β, IL‐6, and IL‐10) in duodenum tissue, serum malondialdehyde (MDA), glutathione peroxidase (GSH‐PX), fecal mucin, secretory immunoglobulin A (sIgA), albumin, lipopolysaccharide (LPS), and antrimethylamine N‐oxide (TMAO) were carried out by enzyme‐linked immunosorbent assay (ELISA) kits from Shanghai Ruifan Biotechnology Co., Ltd., China, by following the company's instructions. Similarly, blood and fecal urea nitrogen (BUN), ammonia, and hydrogen peroxide (H_2_O_2_) were computed using commercial kits (Nanjing Jiancheng Bioengineering Institute, Nanjing, China) as per the instructions of the company. Furthermore, total antioxidant capacity (TAOC) in blood serum was detected by a commercial product of Solrabio Life Science Beijing, China. For fecal/intestinal digesta contents, the feces and intestinal digesta were diluted with double‐distilled water at the ratio of 1:5 (weight/volume), homogenized, and centrifuged (12,000 rpm, 5 min, 4°C). The obtained supernatant was used to detect different contents by following the kit instructions. Digesta pH was measured by inserting the sterile glass electrode of a pH meter (Thermoscientific™ Eutech Elite pH Spear) to a tube containing digesta, and the values were noted down.

### 2.9 Determination of mRNA expression

The expressions of mRNA were determined by quantitative real‐time PCR (q‐PCR). For RNA extraction, intestinal tissues stored at −80°C were taken. Total RNA was extracted from intestinal sections (duodenum, jejunum, and ileum) using RNA extraction kit (Guangzhou Magen Biotechnology Co., Ltd, China) as per the manufacturer's instructions, and then determined using a Nanodrop spectrophotometer. Complementary DNA (cDNA) was synthesized by using 2 μg of total RNA by treating with DNase I (Takara Bio Inc., Shiga, Japan) to obtain a final volume of 20 μL by using Random Primer 9 (Takara Bio Inc., Shiga, Japan) and M‐MLV Reverse Transcriptase (Promega, Madison, WI, USA) by following the protocol of the company. Then, total cDNA was mixed with antisense primers, SYBR green Real‐Time PCR master Mix and Nucleic acid‐free water. The qPCR reaction had a final volume of 20 μL. The qPCR was carried out with the Applied Biosystems QuantStudio 3 Real‐Time PCR System (Thermo Fisher Scientific, USA). GAPDH was used as control, and the relative gene expression of mRNA was calculated by 2^−ΔΔ*Ct*^. The primer sequences used for PCR are provided in Table 1.

**TABLE 1:** Primer sequences used for PCR.

Gene	Forward primer sequence (5′‐3′)	Reverse primer sequence (5′‐3′)
GAPDH	GGAGCGAGACCCCACTAACA	CGGAGATGATGACCCTTTTG
MUC‐2	ATGCCCACCTCCTCAAAGAC	GTAGTTTCCGTTGGAACAGTGAA
Claudin‐1	TGGATGGCTGTCATTGGG	GTGTTGGGTAAGAGGTTGTTTTC
Occludin	CAGCCTTCTGCTTCATCG	GTCGGGTTCACTCCCATTA
ZO‐1	GGGAAAACCCGAAACTGAT	CGCCCTTGGAATGTATGTG
IL‐1β	TGTGCTCTGCTTGTGAGGTGCTG	CCCTGCAGCTGGAGAGTGTGGA
IL‐6	TAGTCCTTCCTACCCCAATTTCC	TTGGTCCTTAGCCACTCCTTC
IL‐8	CACCCTCTGTCACCTGCTCAA	ATGGCGCTGAGAAGACTTGGT
IL‐10	GCTCTCTGAAGAAAGCTGCAC	CACTTTCCCATCTTCATCATCA
TNF‐α	CCGCACACTCAGATCATCTTCT	GCTACGACGTGGGCTACAG

### 2.10 Detection of concentration of lactate and SCFAs in digesta

The determination of SCFA and lactic acid concentrations were done via HPLC (high‐performance liquid chromatography). In brief, the fecal and jejunal contents were taken out from a refrigerator, thawed at 4°C, and mixed thoroughly. Around 0.2 g of the contents from each sample was diluted with double‐distilled water at the ratio of 1:5 (w/v) and mixed using a vortex for around 20 min to break all the feces. The mixture was centrifuged for 15 min, 12,000× *g* at 4°C. Around 400 μL of the supernatant was extracted via pipette after centrifugation. By the use of a disposable syringe, the supernatant was filtered via a 0.22 μm filter, placed in a glass sample bottle, and injected into a glass column of 4.6 × 250 mm dimension. As per the protocol of the company and the parameters of the machine, the test was carried out.

### 2.11 16S rRNA gene sequencing analysis

After 21 days of feeding, fresh fecal samples from four mice of control and encapsulated groups were collected on a well‐sterilized 1.5 mL tube twice a day for 5 days, placed on liquid nitrogen, and stored at −80°C until DNA extraction. 16S rRNA sequencing was performed at Beijing Novogene Co., Ltd. The steps and procedure of the experiment are described in the previous article (F. Zhang et al., 2020). Briefly, CTAB/SDS method was used to extract genome DNA. The purity and concentration of obtained DNA were examined by using 1% agarose gel and diluted to 1 ng/μL using sterile water. The V3–V4 region of the 16S rRNA gene was amplified by using universal primer 338F and 806R with a barcode. All PCR reactions were carried out with Phusion® High‐Fidelity PCR Master Mix (New England Biolabs). Then, the PCR products were detected on 2% agarose gel electrophoresis. The obtained mixture was purified with Qiagen Gel Extraction Kit (Qiagen, Germany). The sequencing library was generated using the TruSeq® DNA PCR‐Free Sample Preparation Kit (Illumina, USA), and quality was assessed using a Qubit@ 2.0 Fluorometer (Thermo Scientific) and Agilent Bioanalyzer 2100 system. The library was sequenced on an Illumina NovaSeq platform, and 250 bp paired‐end reads were generated.

### 2.12 Bioinformatics and statistics

First, raw tags were obtained by using the software flash (version 1.2.7, http://ccb.jhu.edu/software/FLASH/) (Magoč & Salzberg, 2011), and high‐quality clean tags were obtained by using QIIME (version 1.9.1, http://qiime.org/scripts/split_libraries_fastq.html) (Bokulich et al., 2013). Chimera sequencing was detected using UCHIME Algorithm, (http://www.drive5.com/usearch/manual/uchime_algo.html) and effective tags were obtained by the removal of chimera sequences (Edgar et al., 2011). Sequences analysis was performed by using Uparse software (Uparse version 7.0.10, http://drive5.com/uparse/). Sequences with ≥97% similarity were assigned to the same OTUs (Edgar, 2013). As per the Mothur method and SILVA (http://www.arb‐silva.de/) database (Quast et al., 2013), representative sequences for each OTU were annotated with taxonomic information. OTUs abundance information was normalized using a standard of sequence number corresponding to the sample with the least sequences. The alpha and beta diversities of the gut microbiota were analyzed by QIIME software. Graphpad Prism 8.0.1 (Chicago, IL, USA) was used for statistical analysis. All the experimental results are expressed as means ± standard error of the mean (SEM). Methods of statistical analyses were chosen as per the design of each experiment and are mentioned in the figure legends, and *p*‐values of <.05 were considered statistically significant.

## 3 RESULTS

### 3.1 The viability of *L. paracaesi* is enhanced in vitro through its encapsulation

**FIGURE 1:**
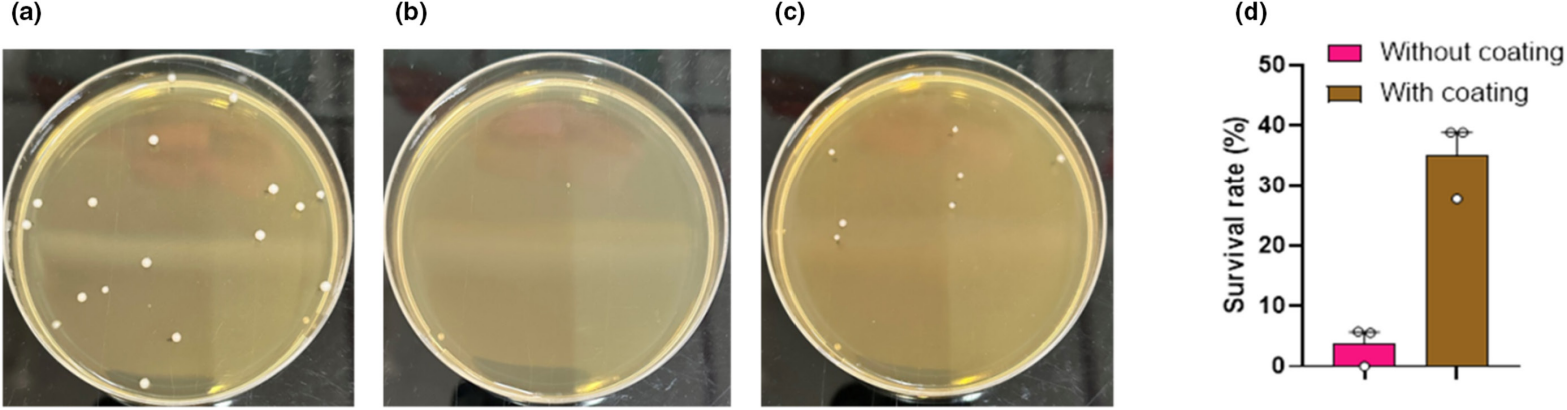
In vitro survival test of coated bacteria. In vitro survival rate of *Lactobacillus paracasei*: microcapsule before exposure to artificial gastric and intestinal juices (a), mixture of probiotics and coating material after exposure (b), microcapsule after exposure (c), and comparison of survivability with and without coating (d).

The use of different materials to encapsulate probiotics has been found to offer protection against the harsh conditions of the gastrointestinal tract. In this study, both free and encapsulated probiotic cells were subjected to simulated gastric and intestinal fluids. Results showed that after approximately 3 h, there was a decrease in the number of nonencapsulated probiotics compared to encapsulate ones (Figure 1a–c). The use of polyacrylate resin as an encapsulating material increased the survival rate of the probiotics; without coating the survival rate was only 5.56%, 0%, and 5.57% while after coating, the survival rate increased to 38.89%, 27.78%, and 38.89%.

### 3.2 Encapsulated probiotics increased fecal weight and moisture content

**FIGURE 2:**
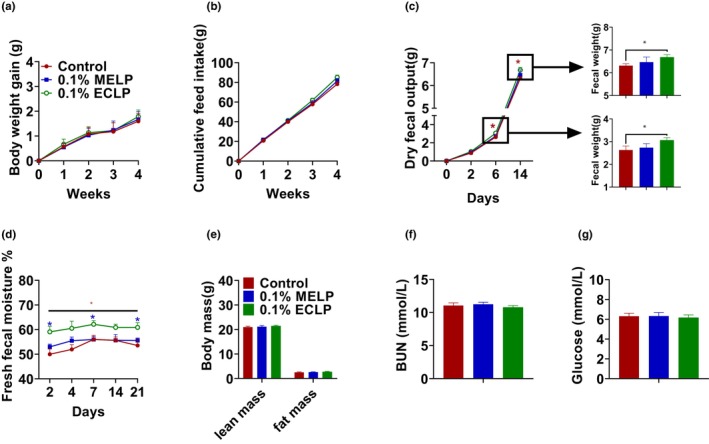
Effect of encapsulated probiotic on growth performance, fecal output, and metabolism: Body weight gain (a), Cumulative feed intake (b), Dry fecal weight (c), Fecal moisture %, * represents significant difference of 0.1% ECLP with control and * with 0.1% MELP (d), Body composition analysis by QMR (e), Blood urea nitrogen (BUN) (f), Glucose (g). The data are presented as the mean ± SEM. *p < .05, **p < .01, (*n* = 8–10 per group). Here, * represents a significant difference in ECLP group compared to control group and * represents a significant difference compared to MELP group. Control group supplemented with basal feed without additives, 0.1% MELP group supplemented with 0.1% of a mixture of encapsulating material and *Lactobacillus paracasei* and 0.1% ECLP group supplemented with 0.1% of encapsulated *L. paracasei*.

The effect of encapsulated and un‐encapsulated *Lactobacillus* was studied on growth performance and fecal parameters. There were no significant differences (*p* > .05) in a body weight gain or feed intake throughout the experiment (Figure 2a,b). Dry fecal weight was significantly increased on the 6th and 14th days of the experiment in encapsulated group (Figure 2c). In addition, the fecal moisture percentage was also remarkably raised in the encapsulated group (*p* < .05) compared to the control (Figure 2d) from the 2nd day of the experiment. Similarly, on the 28th day, QMR was carried out, but no difference was observed in the lean and fat mass among the groups (Figure 2e). Blood urea nitrogen (BUN) and blood glucose also showed no notable difference (Figure 2f,g).

### 3.3 Encapsulated probiotics improved overall histomorphometric parameter and barrier function of the intestine

**FIGURE 3:**
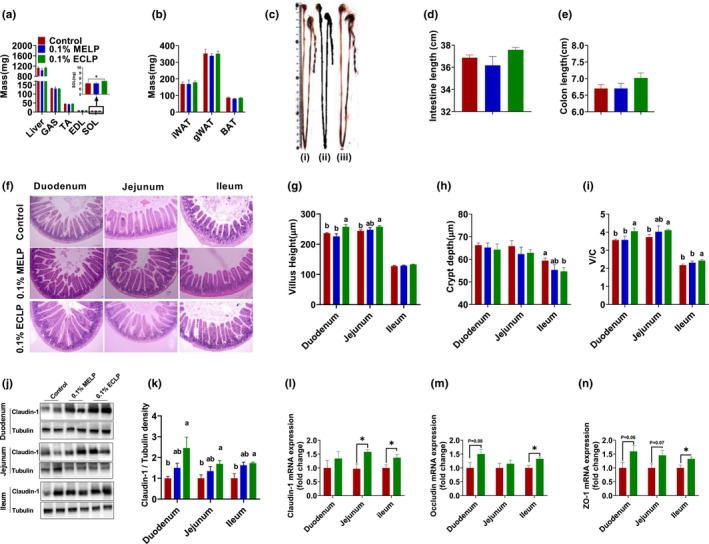
Effect of encapsulated probiotic on histomorphometry, specific organ, and intestinal barrier function: Liver, muscle (GAS, TA, EDL, SOL) mass (a), mass of adipose tissue (b) (*n* = 8–10 per group), Representative image of the intestine (c), supplemented with control feed (i) 0.1% MELP group supplemented with 0.1% of a mixture of encapsulating material and *Lactobacillus paracasei* (ii) 0.1% ECLP group supplemented with dietary encapsulated *L. paracasei* (iii), intestinal length (d), Colon length (e), Representative histology of intestinal section, stained with hematoxylin and eosin (h and e), Bar 100 μm (f), The average: Villus height (g), Crypt depth (h), Ratio of Villus height to Crypt depth (i), The protein expression of Claudin‐1 in the different tissue of intestine (j) (k), mRNA expression of Claudin‐1 (l), Occludin (m), ZO‐1 (n) quantified against housekeeping gene GAPDH on intestinal tissues (*n* = 6–8 per group). The data are presented as the mean ± SEM, *p < .05, **p < .01, different letters above error bars (i.e., ±SE) indicate significant differences (*p* < .05) among groups. Control group supplemented with basal feed without additives and 0.1% ECLP group supplemented with 0.1% of encapsulated *L. paracasei*.

The liver, muscle, and adipose tissue organ indices are displayed in Figure 3a,b. Liver and part of skeletal muscle; gastrocnemius (GAS), extensordigitorum longus (EDL), and Tibialis anterior (TA) did not show any notable difference in their weight. Interestingly, the soleus (SOL) mass was increased significantly (*p* < .05) in encapsulated group compared to control. Additionally, the weight of white adipose tissue (WAT), that is, inguinal (iWAT) and gonadal (gWAT), as well as the weight of brown adipose tissue (BAT) showed no significant differences among the groups. To investigate the morphological change and structure, initially the intestinal and colon length were measured. The length was found to be numerically higher in ECLP group but had no significant difference (*p* > .05) (Figure 3c–e). Using H&E staining, the histomorphological study was made on different sections of the intestine (Figure 3f). All the intestinal sections showed normal tissue. Villus height, crypt depth, and the ratio of villus height to crypt depth (V/C) in the three evaluated intestinal segments (duodenum, jejunum, and ileum) are shown in Figure 3g–i. The addition of encapsulated *Lactobacillus* increased V/C in the duodenum and ileum compared to the control and un‐encapsulated group (*p* < .05). In addition, the dietary treatment of microcapsule increased the villus height significantly (*p* < .05) in the duodenum and jejunum with respect to control. Meanwhile, compared to the un‐encapsulated group, encapsulated group raised villus height in duodenum. Furthermore, the ileal villus height was increased numerically (*p* = .08), and crypt depth was decreased significantly (*p* < .05) in the encapsulated group. But, no notable improvement in intestinal morphology was observed in the un‐encapsulated group, as there was no remarkable change in villus height, crypt depth, or their ratio compared to control. Furthermore, when we studied the effects of microcapsules by WB on the intestinal barrier in the duodenum by which is crucial for tissue morphology and absorption function, we noted an elevation in Claudin‐1 in the encapsulated group (Figure 3j,k).

The above results clearly show that the un‐encapsulated group had no differences in fecal output, body mass, tissue index, intestinal morphology, and intestinal barrier when compared to the control group, but the encapsulated group had significant changes in fecal output and improved intestinal morphology and barrier function when compared to both control and un‐encapsulated groups. One possible explanation for this could be the use of a lower amount of probiotics, which may have resulted in reduced survivability during passage through the gastrointestinal tract.

Ultimately, it concludes that supplementing with encapsulated *L. paracasei* improves intestinal histomorphometry and fecal quality. Thus, we used only control and encapsulated group mice samples for further investigation, if the microcapsule has other beneficial impacts on gut health. Initially, we observed an increase in the mRNA expression levels of tight junction proteins including claudin‐1, occludin, and zonula occludens‐1 (ZO‐1) in the duodenum, jejunum, and ileum sections in the encapsulated group compared to the control, as shown in Figure 3j–l determined by q‐PCR.

### 3.4 Encapsulated probiotics improved immunity and antioxidant capacity

**FIGURE 4:**
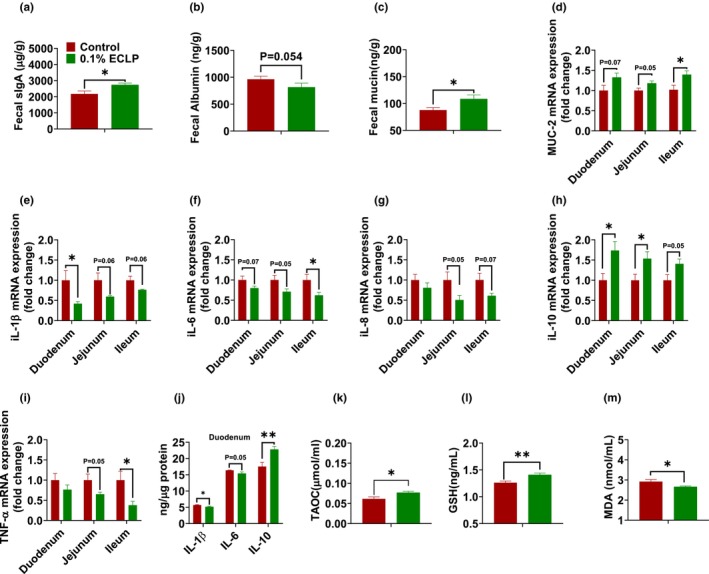
Effect of encapsulated probiotics on immunity and antioxidant capacity. Fecal sIgA (a), Fecal albumin (b), Fecal mucin (c), mRNA expression of MUC‐2 (d), IL‐1β (e), IL‐6 (f), IL‐8 (g), IL‐10 (h), and TNF‐α (i) quantified against GAPDH, Level of inflammatory factor (IL‐1β, IL‐6, and IL‐10) in duodenum (j) TAOC (k), GSH‐Px (l), MDA (m). Here, single bar for feces represents the feces collected on third week. The data are presented as the mean ± SEM. *p < .05, **p < .01. (*n* = 6–8 per group). Control group supplemented with basal feed without additives and 0.1% ECLP group supplemented with 0.1% of encapsulated *Lactobacillus paracasei*.

Fecal secretory immunoglobulin A (SIgA) and albumin contents were quantified as an indicator of the intestinal barrier. Encapsulated *Lactobacillus* raised the quantity of SIgA (*p* < .05) and lowered the level of albumin (*p* = .054) (Figure 4a,b). Mucin level in feces was also analyzed to investigate the effect on a gut barrier function, which was significantly upraised in our encapsulated group (Figure 4c). An increase in mucin contents denotes that encapsulated *Lactobacillus* enhanced the mucin‐related gene expression in the intestinal region. So, analysis of mucin gene; mucin‐2 was quantified by q‐PCR on the different sections of the intestine. There was an increase in Muc‐2 mRNA expression in all the sections of the intestine and a significant increase was found in the ileum section (Figure 4d). To examine the inflammatory cytokines, the mRNA expression of interleukin 1β (IL‐1β), interleukin 6 (IL‐6), interleukin 8 (IL‐8), tumor necrosis factor alpha (TNF‐α), and interleukin 10 (IL‐10) in the different tissue of the intestine were detected by qRT‐PCR. We observed the level of IL‐1β, IL‐6, IL‐8, and TNF‐α were significantly or numerically lower in the duodenum, jejunum, and ileum (Figure 4e–i). IL‐1β level was significantly decreased in the duodenum segment and was also reduced in the jejunum (*p* = .06), and ileum (*p* = .06). In addition, the expression of IL‐6 was reduced in the duodenum (*p* = .07), jejunum (*p* = .05), and ileum (*p* < .05). We also found that IL‐8 expression was downregulated in the duodenum, jejunum (*p* = .05), and ileum (*p* = .07). A significant decrease (*p* < .05) in TNF‐α mRNA expression was observed in the ileum section, but no significant difference was found in the duodenum and jejunum (*p* = .05). Additionally, the consumption of encapsulated *Lactobacillus* increased the level of IL‐10 compared to those in the control group, suggesting the improvement of inflammation. Similarly, an ELISA test was conducted to confirm the presence of inflammatory cytokines in the duodenal tissue. The results of the test indicated a significant decrease in IL‐1β (*p* < .05) and IL‐6 (*p* = .05), as well as an increase in IL‐10 (*p* < .05). As shown in Figure 4k,l, treatment of 0.1% encapsulated *Lactobacillus* elevated the level of total antioxidant capacity (TAOC) and glutathione peroxidase (GSH‐Px) in blood serum. In addition, we observed the reduction of malondialdehyde (MDA) level (*p* < .05) in the encapsulated group (Figure 4m).

### 3.5 Encapsulated probiotics regulated and promoted microbial metabolites

**FIGURE 5:**
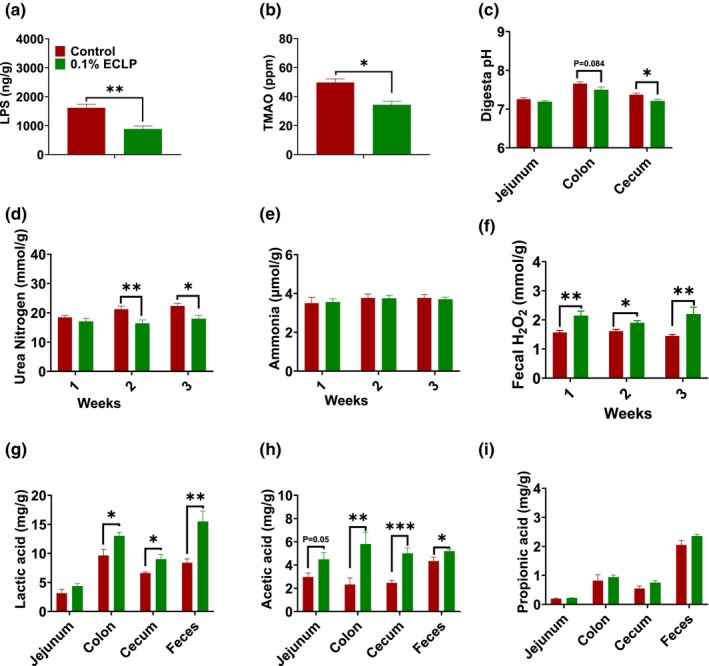
Effect of encapsulated probiotic on microbiome metabolites: Fecal LPS (a), Fecal TMAO (b), Digesta pH (c), Fecal Urea Nitrogen (d), Fecal Ammonia (e), Fecal H_2_O_2_ (f), Lactic acid (g), Acetic acid (h), Propionic acid (i). Here, single bar for feces represents the feces collected on third week. The data are presented as the mean ± SEM. *p < .05, **p < .01, ***p < .001 (*n* = 6–8 per group). Control group supplemented with basal feed without additives and 0.1% ECLP group supplemented with 0.1% of encapsulated *Lactobacillus paracasei*.

The feces stored in the refrigerator were used to determine the effect on the different fecal indices for microbiota study. The fecal lipopolysaccharide (LPS) and trimethylamine‐N‐oxide concentration (TMAO) were decreased (Figure 5a,b) in encapsulated *Lactobacillus* group compared to control with a significant difference. In addition, the pH of digesta was reduced in the jejunum, colon (*p* = .08), and cecum (*p* < .05) (Figure 5c). The value of urea nitrogen in feces was determined for 3‐week period. We found the values were decreased for the whole period. For the first week, no significant difference was seen, but for the second and third weeks, there was a significant reduction (*p* < .05) in its level (Figure 5d). Similarly, the level of fecal ammonia did not show any considerable variation for 3 weeks (Figure 5e). The effect of encapsulated *Lactobacillus* on peroxide production revealed an elevation in H_2_O_2_ production for the same period (Figure 5f). To show the additional potential of encapsulated *Lactobacillus* to influence the gut microorganisms, the SCFA level was examined. Figure 5g–i shows lactic acid and SCFAs concentration in digesta of jejunum, colon, and cecum along with feces. Encapsulated *Lactobacillus* increased the levels of lactate and acetate in colon digesta, cecum digesta, and feces significantly. But, in the jejunum, the rise in lactate and acetate was seen but was not significant. Furthermore, the levels of propionic acid were close in both groups but were slightly higher in the treatment group.

### 3.6 Encapsulated probiotics altered fecal microbiota and their predicted function

**FIGURE 6:**
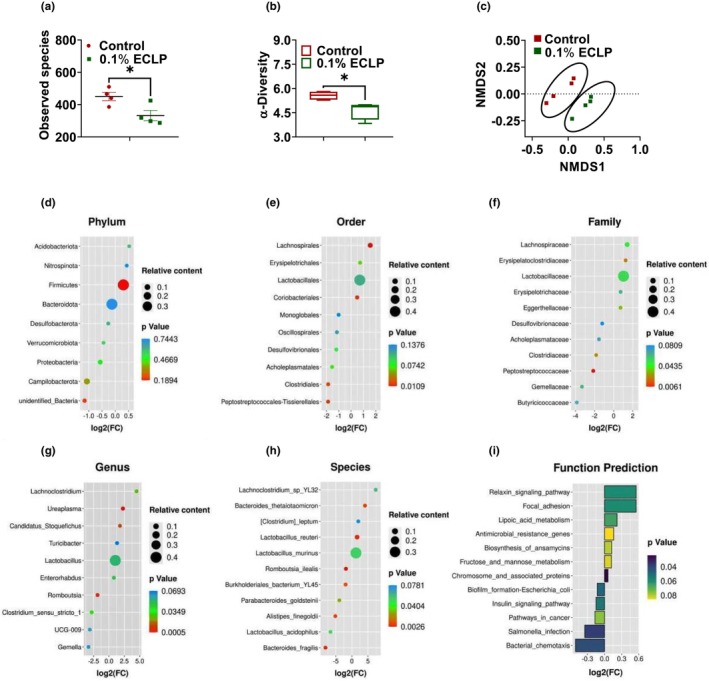
Effect of encapsulated probiotic on modulation of microbiota: Observed species (a), Shannon index (b), Nonmetric multidimensional scaling (NMDS) (c), Composition of gut microbiota by using KEGG enrichment analysis: Phylum (d), Order (e), Family (f), Genus (g), Species (h) where the size of the bubble represents the relative abundance of bacteria, the *x*‐axis label shows log2 (FC) that represents fold change, which was calculated as first, the relative content of bacteria in the control group was divided by our treatment group to get first value (FC). By using excel, log2 (FC) was calculated to get log2 (Fold change). Bacteria that are up‐regulated relative to the control has a positive log2 FC value, and downregulated relative to the control has a negative log2 FC value, left *y*‐axis label represents the bacteria at a different level and a different color on the right side shows significance (*p* value), Prediction of metabolic function at KEGG (level 3) (i), left *y*‐axis shows different predicted function, *x*‐axis shows the log2 (FC) that represents fold change. Control group supplemented with basal feed without additives and 0.1% ECLP group supplemented with 0.1% of encapsulated *Lactobacillus paracasei*.

To study the effects of encapsulated *Lactobacillus* on fecal microbiota, feces were analyzed by 16S rRNA sequencing. We investigated that the consumption of encapsulated *Lactobacillus* decreased (*p* < .05) the observed species (Figure 6a). Similarly, the microbial diversity within the samples was analyzed by using the Shannon index, which determines the richness and evenness of microbial communities. We observed a significant reduction in the Shannon index (*p* < .05) in the encapsulated group (Figure 6b). We used nonmetric multidimensional scaling (NMDS) plots to find the relationship between samples and species (beta diversity) and observed a distinct clustering of the control and treatment group samples (Figure 6c).

Similarly, to evaluate the effect of encapsulated *Lactobacillus* on microbial composition, bacteria on different levels were analyzed. A Kyoto Encyclopedia of Genes and Genomes (KEGG) enrichment analysis was used to express the enrichment of the microbiome on a different level (Figure 6d–h). On the phylum level, no significant differences were observed between the control and encapsulated group, but it was observed that *Firmicutes* and *Bacteroidota* followed by *Campilobacterota* were dominant over other phyla. The result also revealed that consumption of encapsulated *Lactobacillus* elevates the relative abundance of *Firmicutes* (0.3757 vs. 0.4975, *p* = .18) and reduces the *Bacteroidota* (0.3761 vs. 0.3469, *p* = .72) (Figure 6d). In addition, we analyzed the dominant bacteria in order and family level. The proportion of *Lactobacillales* order and *Lactobacillaceae* family was dominant and higher in the encapsulated group than in the control. We also revealed a significant increase in *Lachnospirales* order and *Lachnospiraceae* family in encapsulated *Lactobacillus* group (Figure 6e,f).

*Lactobacillus* and *Bacteroides* were dominant bacteria at the genus level. The relative abundance of *Lactobacillus, Turicibacter*, and *Lachnoclostridium* was elevated in the treatment group, and *Bacteroides* was slightly reduced. There was a statistically significant reduction of *Romboutsia* in the treatment group (Figure 6g). Additionally, we also observed a reduction of harmful bacteria like *Streptococcus, Helicobacter, Corynebacterium*, etc. without statistical significance. At the species level, *Lactobacillus* sp. was dominant over others. *Lactobacillus murinus* was the dominant species in both groups, but it was significantly higher in the encapsulated group. Similarly, *Lactobacillus reuteri*, and *Bacteroides thetaiotaomicron* were markedly elevated in the treatment group, meanwhile *Lactobacillus acidophilus*, *Bacteroides fragilis*, and *Romboutsia ilealis* were significantly reduced (Figure 6h).

To further analyze the relative difference between the control and encapsulated *Lactobacillus* in terms of function prediction of microbiota in feces, a histogram with FC and *p* value was used to analyze their KEGG pathway based on 16s rRNA sequencing. The feces of mice had shown that encapsulated *Lactobacillus* group has a higher relative abundance of microbiota involved in fructose, mannose, and lipoic acid metabolism. We found those microbiomes related to antimicrobial resistance genes and biosynthesis of ansamycins were enriched. However, the pathways in cancer, salmonella infections, bacterial chemotoxins, and biofilm formation in *Escherichia coli* were reduced in the treatment group (Figure 6i).

## 4 DISCUSSION

The purposes of microencapsulation are to mitigate the poor viability of probiotics due to the harsh environment on the upper part of the GI tract, and to release probiotics at a controlled rate on the lower part for its beneficial action to the host. Previously, several microencapsulation methods and materials have been studied on different strains of probiotics to analyze their viability. Alginate‐pectin microgel (Zhuge et al., 2020), chitosan‐alginate (Lohrasbi et al., 2020), cellulose sulfate (Gunzburg et al., 2020), etc., as an encapsulating material protected different bacterial and fungal strains of probiotics from unfavorable gastrointestinal environment and improved the survival of the bacterial cell, intestinal delivery and release resulting to several health benefits. Moreover, some materials like pectin‐encapsulated probiotics did not enhance the effect of probiotic supplementation (Lee et al., 2019). In our study, polyacrylate resin was used as encapsulating material that can hold its structure at lower pH and release probiotic strain completely on the hindgut. The moisture content in feces, which determines the softness or firmness, was significantly increased throughout the experiment. Our results were consistent with earlier studies on probiotic species (Gan et al., 2020; Saw et al., 2019). The production of lactic acid (Saw et al., 2019) may be the reason for a higher moisture content that helps to alleviate constipation and induce bowel movement.

The present study revealed that probiotic *Lactobacillus* species could improve intestinal morphology as we observed an increase in the V/C ratio on different intestinal tissues. They are the standard index for intestinal health and indicate enhancement in digestion and absorption by increasing the epithelium surface layer (Celi et al., 2017). Similarly, the intestinal epithelium layer represents the most important barrier against pathogenic molecules and bacteria. The integrity of the intestinal epithelial cell layer is maintained by adherens junctions, tight junctions (TJ), and desmosomes. Claudin, occludin, and zonula occludens (ZO‐1) are majorly studied tight junction proteins (Schneeberger & Lynch, 2004) and their expression was increased in the intestinal tissues. Our result is consistent with other studies on different probiotic species (Bao et al., 2021; Yi et al., 2017). Numerous studies strongly suggest that gut microbiota can influence TJ expression and assembly, and hence regulate transepithelial permeability (Allam‐Ndoul et al., 2020).

The gut microbiota contributes to host physiology by producing a wide range of metabolites. LPS, also called endotoxins, and TMAO, are intestinal microbiome‐derived toxins correlated with inflammation, cardiovascular disease (CVD), and other diseases on the host (Yamashita et al., 2021). Reduction of their level in the encapsulated group suggests that *L. paracasei* reduces the toxins production and the expression of markers of inflammation (Wang et al., 2019). Similarly, short chain fatty acids (SCFAs) are other key metabolites of microbiota in the colon. Lactate, short‐chain hydroxyl‐fatty acid, is produced by several bacterial species and converted to SCFA by lactate fermenting bacteria (Russell et al., 2013; Silva et al., 2020). The three monocarboxylic short‐chain organic acids, lactate, acetate, and propionate, can maintain immune and intestinal homeostasis by downregulating the proinflammatory response in intestinal epithelial cells, showing antimicrobial and anti‐inflammatory effects (Iraporda et al., 2015; Tan et al., 2014). Our findings investigated a rise in the SCFA and lactate value in colonic, cecal, and fecal content, which was consistent with several other probiotic species of *Lactobacillus*, *Bifidobacterium*, and *Streptococcus* that produce lactate and SCFA (Markowiak‐Kopeć & Śliżewska, 2020). The modulation of microbiome metabolites is associated with a change in gut microbiota.

Probiotics have a vital role in maintaining the gut microbiota by increasing the population of beneficial bacteria and reducing the pathogens by competing with them (Kechagia et al., 2013; Plaza‐Diaz et al., 2019). Previously, several studies were conducted on the impact of different strains of probiotics, including *Lactobacillus*, and it is reported that the probiotic modulates the microbiome on the different intestinal segments and controls the microbes ecosystem (Azad et al., 2018). Our study also found that the supplementation of encapsulated *L. paracasei* changed the microbiome population. The treatment of encapsulated probiotic limited microbiome to predominantly *Lactobacillus* genus (0.4000 vs. 0.1952) and *L. murinus* species (0.3194 vs. 0.1314), which could be the reason for the decrease in richness and diversity as found in our study. Moreover, we observed a clear difference between the control and encapsulated probiotic‐treated group, that is, beta diversity, which illustrates *L. paracasei* affects the microbiota in mice.

Similarly, an increase in *Firmicutes* and a decrease in *Bacteroidetes* phyla are correlated with increased absorption of nutrients (Jumpertz et al., 2011), which indicates that the supplementation of encapsulated *Lactobacillus* improves digestion by improving absorption. We also found upraised value in *Firmicutes* to *Bacteroidetes* ratio (0.99 and 1.43) after supplementing the encapsulated probiotic, which is believed to be the marker for obese animals. However, we did not find any difference in weight gain in mice between those groups. A recent study has presented that this biomarker is still difficult to associate with the weight and health of an individual (Magne et al., 2020). Interestingly, we observed a significant increase in soleus weight that could be due to the variation in the gut microbiota and metabolites produced by them that influence the skeletal muscle mass (Lahiri et al., 2019). But, for an accurate conclusion, further research is necessary. Raise of *Lachnospiraceae* family in encapsulated group could be beneficial to host as it is chiefly responsible for producing short‐chain fatty acids (Pan et al., 2020).

Genus *Lactobacillus*, belonging to *Firmicutes* phylum, was enriched in the treatment group (*p* = .05), which exert a positive effect on the host's health by maintaining immune homeostasis, improving gastrointestinal barrier function, and suppressing proinflammatory cytokines (Azad et al., 2018). Some species under the *Lactobacillus* genus also can produce several inhibitory substances, including H_2_O_2_, that may limit the growth of pathogens to protect intestinal mucosa by strengthening toxic oxidation (Vieco‐saiz et al., 2019), that could be another cause for the decrease in species richness in the encapsulated group. *L. murinus* relatively and significantly increased in the treatment group. Lebovitz & Theus, 2019 have reported the applications of *L. murinus* from various studies (Lebovitz & Theus, 2019) and outlined that it has a beneficial effect on the host, including antimicrobial production, antagonist against pathogens, intestinal barrier, and can be developed as a potential probiotic. *B. thetaiotaomicron* helps in Carbohydrate metabolism, lipid metabolism, and enervates the production of proinflammatory cytokines, and finally helps to strengthen the host‐microbiome ecosystem (Jandhyala et al., 2015). As per the function prediction, the pathway associated with the antimicrobial resistance gene was increased. Biosynthesis of antibiotics may be the reason for the enrichment of the antimicrobial resistance gene. Here, the pathway related to the synthesis of ansamycins (antibiotic) was also enriched. Pathway associated with lipoic acid metabolism may elevate host antioxidant properties and anti‐inflammation (Moura et al., 2015).

Epithelial cell of the intestine establishes a physical and chemical barrier for preventing antagonism between immune cells of host and gut microbes to protect the mucosa from inflammation (Okumura & Takeda, 2017). SIgA is an abundant antibody class found in the intestinal lumen, illustrated as the first line of defense to protect the intestinal epithelium from pathogens and enterotoxins and has a key role in immune protection, which was upraised in the feces of encapsulated groups (Mantis et al., 2011). Similarly, the increased mucin level and MUC‐2 mRNA expression suggests that the encapsulated group could protect and safeguard the GI tract, as mucin is essential for epithelial lubrication, and MUC2 covers the intestinal tract and protects it from pathogens (Johansson & Hansson, 2016; Kawakami et al., 2020; Okumura & Takeda, 2017). Cytokines have the function to balance the intestinal immunity in the host. Intestinal Infections lead to inflammation that raises several proinflammatory factors (Chen et al., 2018). Li et al., 2016 described that supplementation of *L. acidophilus* against *E. coli* decreased the secretion of proinflammatory cytokines and increased the secretion of anti‐inflammatory cytokines compared to control, indicating probiotics can act as an anti‐inflammatory supplement (Li et al., 2016). In our current study, the supplementation of encapsulated *L. paracasei* decreased the expression of TNF‐α, IL‐1β, IL‐6, and IL‐8 and increased the expression of IL‐10 in different intestinal tissues. Our results are in line with the earlier studies with other probiotics (Chen et al., 2017; Li et al., 2019; Pan et al., 2020). Thus, we can suggest that encapsulated *L. paracasei* can show anti‐inflammatory effects and protect the gut. But, interestingly, there was a difference in the mRNA expression within a tissue. So, further studies are needed to draw a specific conclusion from this.

It is also stated that when enteric commensal bacteria contact gut epithelia, reactive oxygen species are rapidly generated (ROS) (Jones et al., 2012; Shandilya et al., 2022). Higher production of reactive oxygen species (ROS) than antioxidants leads to oxygen stress in host health. Due to such imbalance, there is a disturbance in a cell leading to damage of DNA, lipids, and proteins. During ROS production, several antioxidant enzymes like superoxide dismutase (SOD) and glutathione peroxidase (GPx) defend against oxidative stress for balancing the system. However, MDA activity is increased during oxidative damage (Mishra et al., 2015). In earlier studies, different strains of probiotics including, *Lactobacillus plantarum* KFY02 (Pan et al., 2020), *Bacillus velezensis*, and *Bacillus subtilis* (A. Li et al., 2019), improved the antioxidant level of animals by increasing antioxidant enzymes. An increase in the quantity of TAOC and GSH‐Px and a decrease in the MDA level in our study concludes that our encapsulated probiotics can enhance the antioxidant capacity of animals.

In this study, we developed a novel microcapsule that keeps its structure in gastrointestinal transit and releases the probiotic species completely in the hindgut to improve the overall health performance of the host. We demonstrated that the encapsulation of probiotics with polyacrylate resin could upregulate the anti‐inflammatory cytokines and downregulate proinflammatory cytokines. Additionally, it enhances intestinal barrier function, antioxidant ability, improves intestinal histomorphometry, and promotes microbial metabolites. All these activities are associated with the change in the composition of gut microbiota. Our study suggests that the microcapsule developed can be applied to the commercial production of livestock and poultry. However, more research may be required to improve the efficacy of microcapsules.

## AUTHOR CONTRIBUTIONS

**Ishwari Gyawali:** Conceptualization (supporting); data curation (equal); investigation (equal); methodology (lead); writing – original draft (lead); writing – review and editing (Lead). **Canjun Zhu:** Data curation (equal); investigation (equal); supervision (equal); writing – review and editing (equal). **Guilian Zhou:** Funding acquisition (equal); project administration (equal); resources (equal). **Guli Xu:** Methodology (supporting). **Yuxian Zeng:** Methodology (supporting). **Jincheng Li:** Methodology (supporting). **Jingjing Zhou:** Methodology (supporting). **Gang Shu:** Conceptualization (equal); validation (equal); writing – review and editing (equal). **Qingyan Jiang:** Conceptualization (equal); funding acquisition (equal); investigation (equal); project administration (equal). **Genghui Li:** Methodology (supporting). **Yujun Wang:** Methodology (supporting).

## FUNDING INFORMATION

The project was supported by grant of the Guangdong key research and development program (2019B020218001) and the National Natural Science Foundation of China (32102626), the Local innovative and research teams project of Guangdong province (2019BT02N630), Quality Control for Feed and Products of Livestock and Poultry Key Laboratory of Sichuan Province (NH2021202206).

## CONFLICT OF INTEREST STATEMENT

The authors confirm that they have no conflict of interest.

## ETHICS STATEMENT

Animal care and procedures were performed as per the guidelines and were approved by the Animal Subjects Committee of South China Agricultural University and Department of Science and Technology of Guangdong Province (permission number: SYXK [Yue] 2014‐0136).

## Data Availability

The datasets generated during and/or analyzed during the current study are available from the corresponding author on reasonable request.
